# Primary care performance in a Ugandan rural district: a cross-sectional descriptive study

**DOI:** 10.3399/BJGPO.2024.0105

**Published:** 2025-04-24

**Authors:** Innocent Kabahena Besigye, Robert James Mash

**Affiliations:** 1 Division of Family Medicine and Primary Care, Faculty of Medicine and Health Sciences, Stellenbosch University, Cape Town, South Africa; 2 Department of Family Medicine, School of Medicine, Makerere University College of Health Sciences, Kampala, Uganda

**Keywords:** primary health care, quality care, Uganda, cross-sectional studies

## Abstract

**Background:**

To strengthen primary health care (PHC), there is a need to measure its performance. Global recommendations emphasise patient involvement in the improvement of services.

**Aim:**

To measure primary care performance in one rural Ugandan district.

**Design & setting:**

A cross-sectional survey of Tororo District where the Ugandan Primary Care Assessment Tool (UG-PCAT) was administered across a sample of 51 facilities. There were four levels of health facilities (health centre [HC] II, HC III, HC IV, and general hospital).

**Method:**

A sample of 488 users was obtained from each of the four levels while including all primary care providers and managers at the health facilities. Data were collected in REDCap software, and analysed using Statistical Package for Social Sciences (version 23).

**Results:**

Only 35.1% of users had a strong affiliation with their PHC facility. Overall, the primary care score suggested that performance was acceptable to the majority of users (58.9% rating performance at least acceptable). Ongoing care was rated by users as very poor (<25% of people rating it at least acceptable). Comprehensiveness (services available) was rated poor by users (<50% finding it at least acceptable). Users rated first-contact access and coordination (information systems) acceptable (51–75% finding them at least acceptable). Person-centredness and comprehensiveness (services provided) were rated good by users (>75% rating them as acceptable or more). Providers and users differed significantly (*P* value <0.05) in their scoring across all domains, with providers usually more positive. Performance significantly improved as the PHC level increased (*P* value <0.05).

**Conclusion:**

Primary care performance in the study district was suboptimal. The UG-PCAT identified primary care functions that needed improving and may be a useful tool to measure PHC performance across the region.

## How this fits in

Measurement of primary care performance helps to identify gaps that can be targeted for improvement. No such measurement on core functions of primary care has ever been done in Uganda despite its health system being based on primary health care (PHC). The study evaluates the performance of primary care in a Ugandan district, identifying both areas of strengths and weaknesses. The areas of weakness should be targeted for improvement by both providers and managers.

## Introduction

The 2018 Declaration of Astana re-energised many countries to improve primary healthcare (PHC) performance as a key to achieving universal health coverage.^1^ PHC has three components, multisectoral policy and action, community empowerment, and integrated primary care services with essential public health functions.^2^ Service delivery should be accessible and available, and of a high quality.^3^ High quality PHC is effective, safe, efficient, timely, and delivers on its core functions. These core functions are first-contact access, continuity, comprehensiveness, coordination, and person-centredness. Care should be family and community-oriented, and provided by a competent PHC team that respects the values and culture of the people served.^4^ Such attributes of high quality PHC, should be regularly measured, to identify performance gaps and continuously improve.

Ugandan health policies have traditionally emphasised PHC.^5,6^ However, the country continues to have low investment in health care, particularly PHC. This undermines the strategic vision of the government to protect, promote, and ensure the wellbeing of the people.^7^ As Uganda strives to improve PHC, there is a need for better evidence to guide policy-making. Global recommendations emphasise patient involvement in the measurement and improvement of PHC.^3,8^ The Primary Care Assessment Tool (PCAT) has the potential to fulfil these requirements, and has been used in many countries to measure performance from the perspectives of users, health providers, and managers.^9–11^ Uganda has not measured the core functions of primary care, although a recent study adapted and validated the PCAT for use in Uganda (UG-PCAT).^12^ This study aimed to measure primary care performance in one rural Ugandan district.

## Method

### Study design

This was a descriptive cross-sectional survey.

### Study setting

The study was conducted in Tororo District, which has a total of 58 public sector primary care facilities. Facilities included health centres II (HC II), health centres III (HC III), and health centres IV (HC IV), with the general hospital (GH) forming the apex as a referral centre. HC IIs are community dispensaries and provide ambulatory care, HC IIIs, in addition to ambulatory care, have maternal health services and HC IVs provide emergency surgical and obstetric services. The GH provides comprehensive medical and surgical services to both outpatients and inpatients. No gatekeeping exists to ensure proper utilisation of secondary care. Most primary care providers are nurses and mid-level clinicians, known as clinical officers, are supported by doctors. Most of them are district employees with a few volunteers.

Tororo District is a rural area in Eastern Uganda, with an average total population of 609 117 (2024 national population census) with Jopadhola as the main ethnicity. Subsistence farming is the main economic activity in the district. Infectious diseases form the highest disease burden among the predominantly young population.

### Study population

Users were defined as adult patients, who had used the health facility at least three times in the past year. Very sick patients and those with severe mental problems who were unable to answer the questions were not included. All health workers and managers at the facilities were eligible for inclusion.

### Sample size estimation

A sample of 51 out of the 58 facilities was required, assuming a response proportion of 50%, 5% margin of error, and 95% confidence intervals. A sample of 360 users was required based on a user population of 20 000, 61% giving a good primary care score, and the same parameters.^10,11^ This was rounded up to a target of 400 users to ensure a complete dataset, and stratified equally between the four facility levels of care.

### Sampling procedure

The PHC facilities were selected by simple random sampling. The users’ sample size was further divided equally between the number of selected facilities at that level. The daily attendance register at the selected facilities was used to select patients by systematic random sampling. If the selected patient did not meet the inclusion criteria, the subsequent one was selected maintaining the sampling interval. All primary care providers and managers were selected, without sampling.

### Data collection

The PCAT was originally developed in the US at The John Hopkins Primary Care Policy Center ^13^ and has been adapted and validated for use in multiple countries.^11,14–17^ Before this survey, the PCAT was adapted and validated for Uganda (UG-PCAT) from the South Africa version (Supplementary Table 1).^12,14^

Four research assistants (RAs) were trained by IB to administer the users’ questionnaire via the REDCap application on their mobile devices. The RAs were health-data clerks employed by the district and had no direct contact with patients seen at facilities. The users were selected as they waited to see the clinician and were interviewed after the consultation. A REDCap link to a self-administered UG-PCAT questionnaire was emailed to providers and managers. The researcher closely supervised the RAs during the process of data collection to ensure adherence to the data collection procedures. The data were collected between May and August 2022.

### Data analysis

Collected data were exported from REDCap into the Statistical Package for Social Sciences (version 23.0). Data analysis followed the steps from the PCAT manual.^18^

The strength of affiliation with the facility was categorised into ‘strong’ for those who sought care only from the health facility, ‘moderate’ for those who sometimes sought care from another source, and ‘poor’ for those who sought care from another place or person and were best known there.

Likert scale scores (from 1–4) for each item were combined to compute the overall median score and interquartile range for each domain. The proportion of responders with a median score>3 for each domain was also analysed and interpreted as:<25% very poor performance, 26–50% poor performance, 51–75% acceptable performance, and >75% as good performance.

An overall median primary care score was computed from the combined scores of all the domains measuring the core functions. A median extended primary care score was calculated from the scores of all the domains measured in the UG-PCAT. The domains and primary care scores were compared between responder groups and levels of facilities using the Mann–Whitney and Kruskal–Wallis tests.

## Results

**Figure 1.**
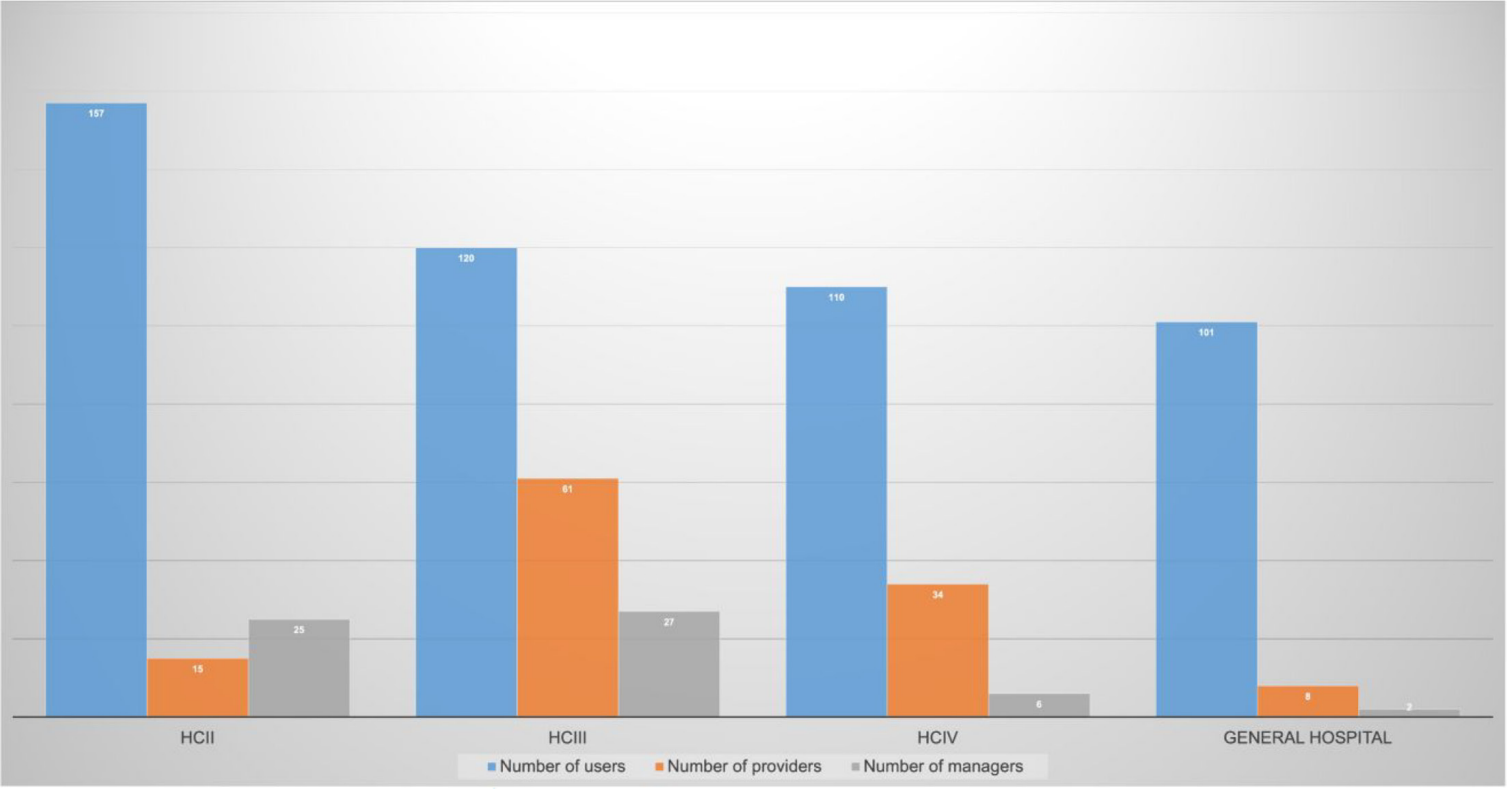
Number of responders per category at each level of primary care

A total of 488 users, 118 primary care providers, and 60 managers participated in the study. Figure 1 shows the number of responders per level of primary care. The mean age of the users was 31.5 years (standard deviation [SD] ± 10.3), the majority were female (85.0%) and Jopadhola speakers. The characteristics of the users and providers are shown in Supplementary Table 2.

Regarding affiliation to the facility, 91/459 users (19.8%) had poor affiliation, 207/459 (45.1%) moderate affiliation, and 161/459 (35.1%) strong affiliation. Table 1 presents the perspective of users on primary care performance. The performance was rated as good for comprehensiveness (services provided), community-orientation, cultural competence, and person-centredness. Users scored first contact (utilisation and access), coordination (information), and family centredness as acceptable. Comprehensiveness (services available), coordination, and the primary care team were scored as poor, and ongoing care as very poor. The overall primary care score was seen as acceptable, but only 58.9% gave a score of >3.

**Table 1.** Proportion of users with >3 scores

	Users’ scores >**3,** *n* (%)
Primary care score	244 (58.9)
Extended primary care score	201 (57.6)
**Domains**	
Community-orientation	460 (96.0)
Person-centredness	413 (91.6)
Comprehensiveness (services provided)	394 (82.4)
Cultural competence	369 (76.6)
First contact (utilisation)	333 (68.8)
Family-centredness	282 (58.0)
Coordination (information systems)	278 (57.4)
First contact (access)	252 (52.0)
Comprehensiveness (services available)	226 (50.1)
Primary healthcare team	229 (47.6)
Coordination (systems)	129 (26.9)
Ongoing care	116 (24.2)

The percentages are out of different total numbers for the domains due to incomplete data.

**Figure 2.**
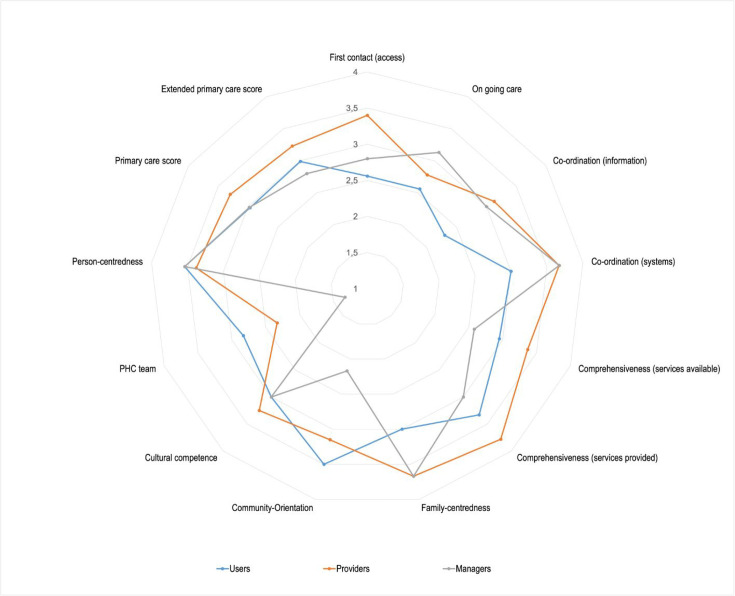
Radar chart of responders’ scores for the primary care domains. PHC = primary health care

Table 2 compares the median scores between responder groups and Figure 2 presents the scores in a radar diagram. For all the primary care domains, there were significant differences between the responders. Providers versus users were statistically different (P values <0.05) in their scores across all domains and providers were mostly more positive about primary care performance. However, they scored community orientation, team composition, and person-centredness significantly lower than the users. There were statistically significant differences (P value <0.05) among most of the domains for users and managers, except for first-contact access (P value =0.257), comprehensiveness (services provided) (P value=0.365), cultural competence (P value=1), and person-centredness (P value=1). Both managers and providers had similar perceptions of the quality of primary care performance with a statistically significant difference (P value <0.05) in only two domains comprehensiveness (both services available and provided) and community orientation. Users rated both the primary care and extended primary care scores as significantly lower than providers. Table 3 shows that the median primary care score significantly improved as the level of primary care increased.

**Table 2.** Comparison of responders’ median primary care scores

Domain	Users’ scores, median (95% CI)	Providers’ scores, median (95% CI)	Managers’ scores, median (95% CI)	Users and managers (*P* value)	User and providers (*P* value)	Managers and providers (*P* value)
First contact (utilisation)	3.0 (2.60 to 3.30)	-	-	-	-	-
First contact (access)	2.56 (2.56 to 2.67)	3.40 (3.40 to 3.60)	2.80 (2.00 to 3.20)	0.257	<0.001	0.006
Ongoing care	2.56 (2.56 to 2.67)	2.78 (2.67 to 3.00)	3.13 (3.00 to 3.50)	<0.001	0.002	0.212
Coordination	2.30 (2.30 to 2.50)	3.13 (3.13 to 3.25)	3.00 (3.00 to 3.25)	<0.001	<0.001	0.063
Coordination (information systems)	3.00 (3.00 to 3.33)	3.67 (3.67 to 4.00)	3.67 (3.67 to 4.00)	<0.001	<0.001	1.000
Comprehensiveness (services available)	2.95 (2.89 to 3.11)	3.37 (3.32 to 3.53)	2.58 (2.16 to 2.79)	<0.001	<0.001	<0.001
Comprehensiveness (services provided)	3.33 (3.33 to 3.44)	3.78 (3.78 to 3.89)	3.00 (2.78 to 3.44)	0.364	<0.001	<0.001
Family-centredness	3.00 (3.00 to 3.33)	3.67 (3.67 to 4.00)	3.67 (3.67 to 4.00)	<0.001	<0.001	1.000
Community-orientation	3.50 (3.50 to 3.67)	3.17 (3.17 to 3.50)	2.17 (2.17 to 2.67)	<0.001	0.027	<0.001
Cultural competence	3.00 (3.00 to 3.20)	3.25 (3.25 to 3.50)	3.00 (3.00 to 3.25)	1.000	0.008	0.051
Primary healthcare team	2.83 (2.83 to 3.00)	2.33 (2.17 to 2.67)	1.33 (1.33 to 1.83)	<0.001	<0.001	0.012
Person-centredness	3.54 (3.54 to 3.69)	3.38 (3.31 to 3.46)	3.54 (3.38 to 3.77)	1.000	<0.001	0.050
**Primary care score**	2.97 (2.95 to 3.00)	3.30 (3.25 to 3.38)	2.98 (2.67 to 3.37)	1.000	<0.001	0.825
**Extended primary care score**	2.99 (2.96 to 3.03)	3.23 (3.17 to 3.33)	2.80 (2.59 to 3.17)	1.000	<0.001	0.050

**Table 3.** Comparison of primary care scores across the levels of care

Healthcare level	Overall median primary care score (95% CI)	Comparison with next higher-level *P* value (95% CI)
HC II	2.63 (2.56 to 2.72)	<0.001
HC III	3.19 (3.13 to 3.30)	0.044
HC IV	3.35 (3.32 to 3.40)	<0.001
General hospital	3.65 (3.60 to 3.70)	-

HC = health centre.

## Discussion

### Summary

Overall, primary care performance was rated as acceptable by users and managers, with providers being more optimistic. Less than half of the users had a strong affiliation with their PHC facility; and this aligns with the poor scores for ongoing care. The following three of the five core functions of primary care were perceived as performing poorly: comprehensiveness (services available); coordination (systems); and ongoing care. First-contact access and utilisation were rated as acceptable, and only person-centredness was seen as good. It was also notable that primary care performance improved as the level of facilities increased, probably owing to better availability of equipment and human resources. However, this may be an indication of inherent inequity within the PHC system, since community members are supposed to receive primary care from health facilities nearest to where they live and work, irrespective of the level.

### Strengths and limitations

The study used a locally validated tool and randomly sampled facilities from the whole district and all primary care levels; thus, the findings should be generalisable to the district. The findings may also be transferable to other districts in Uganda given their similar contexts. Exit interviews may be overly influenced by the experience of services on that day and could also result in a positive Hawthorne effect if providers are aware that such interviews are taking place. Nevertheless, this is the approach recommended by WHO to measure the core functions of primary care and is also feasible.

The study used an equal sample size from each level to enable comparison of performance. However, actual utilisation of primary care by the population may be higher in lower-level facilities as they are more accessible. The overall primary care performance may, therefore, have been even lower than reported.

### Comparison with existing literature

The domains coordination (information systems), comprehensiveness (services provided), family-centredness, cultural competence, and person-centredness were perceived by all the responder groups as performing well (good or acceptable), while the composition of the PHC team was perceived as poor. The users’ perception of the PHC team was better compared with other responder groups’ perception, and may reflect a lack of knowledge regarding the availability of certain team members. All the domains with poor primary care performance in the Ugandan study also had poor performance in the Kenyan study, an indication of similarity in the health system organisation and function.^11^

The users’ perspective was different from providers’ in all 12 domains, and from the managers in seven domains. Their perspective was mostly more negative than the providers. This may be because providers are assessing their own performance using subjective parameters, while users were assessing the performance of others. Managers and providers only differed in five domains. Managers and users had a similar overall primary care score, but providers were significantly more positive. It is possible that managers had a more objective view of primary care performance, while providers needed to believe more in their own performance. Ultimately, the experience and perspective of the users was used as the benchmark. Having the different viewpoints may help stakeholders to reflect on differences and to re-calibrate their own assessment of the strengths and weaknesses. This may particularly be important in feedback to providers and managers, who are responsible for improving primary care performance.

First-contact (access) was just acceptable, and this was similar to findings from other African studies.^9,11,19^ Access is one of the preconditions for ongoing care as users can only continue to use services that are accessible.^20^ This may be the explanation for the rating of ongoing care as poor (providers) and very poor (users). This undermines the well-known contribution of ongoing care to effective use of resources, lower use of out-of-hours services, fewer acute hospital admissions, and lower mortality.^21–23^

The poor scores for comprehensiveness (services available) may be linked to the inadequate composition of the PHC teams and the capability of that team. Underlying this is the low status of PHC in Uganda and underinvestment in human resources. In Uganda, more attention and investment are given to hospital-based care and vertical programmes, despite the espoused health policy on PHC. The available financing is mainly used to develop infrastructure and for recurrent expenditure on essential drugs and medical supplies. There is relatively less investment in the PHC workforce, to ensure they are competent to deliver comprehensive quality PHC. This finding is similar to the Kenyan study, although this was conducted among private sector clinics in Nairobi.^11^ The perceived performance in the Kenyan study may be owing to the model of care, as services relied on a single GP, usually without any postgraduate training, and clinics sent complicated patients to the tertiary hospital. However, in the South African public sector, the PHC team was perceived as having good performance.^9^ The PHC team domain focuses on team composition, which is not a well-developed concept in Uganda and Kenya, when compared with South Africa. For example, in Uganda, there is no clear description of the PHC system and how it should function; while South Africa has a consistent policy focus on PHC service design.^24,25^ Recent PHC re-engineering in South Africa, guided by the WHO framework for health systems strengthening, focused on development of PHC teams to provide comprehensive services.^26,27^

When the findings are compared with other PCAT studies within the African region, there are similarities and differences in performance. In the Kenyan study, most of the domains were perceived to have very poor performance, with only three domains rated as acceptable and one as good.^11^ This undermines the assumption that private health services are inevitably of better quality. All responder groups in the South African study rated most of the domains as having acceptable and good performance, with none rated as very poor.^9^ The users in the South African study rated overall primary care performance as acceptable, similar to Ugandan findings, while in Kenya, it was perceived as poor.^9,11^

The UG-PCAT is currently the only version that measures person-centredness as a core function of primary care. Therefore, the UG-PCAT is able to measure all five of the core functions as defined in the WHO PHC measurement framework.^3^ The WHO suggests that the core functions should be measured by exit interviews at the level of the facility, and the UG-PCAT now provides such a tool. Shortening the tool by focusing on only core primary care domains would make the tool more practical and less time-consuming.

### Implications for research and practice

The UG-PCAT allows the perspective of users on the core functions of primary care to be included in the planning, design, and implementation of primary care services. A future study will evaluate how Tororo district makes use of the findings to improve performance. The findings can enable reform of the model of care, and contribute to systems for improving quality. There may also be implications for the health system inputs such as the workforce and health information system.

A national UG-PCAT survey should be conducted to establish the state of primary care performance across districts, given that the Ugandan health policy is based on PHC. It will also be valuable to measure the primary care performance of the private sector as a key provider of primary care.

The validated UG-PCAT measures all the core functions of quality primary care according to the WHO PHC measurement framework. Further work should be undertaken to develop a regional PCAT that can be used across countries in the WHO Africa region to measure primary care performance.

In conclusion, primary care performance in Tororo District is suboptimal, particularly at the lower levels of PHC facilities. The key stakeholders in primary care hold different views, with providers scoring performance higher than users. The UG-PCAT can measure performance of the core functions of primary care and identify areas that need improvement. Future research will evaluate how the district makes use of the findings to improve performance.

## Data

The authors confirm that the data supporting the findings of this study are available on reasonable request from the authors.
